# Administration of Intravenous Lipid Emulsion for Dextromethorphan Poisoning with Serotonin Syndrome: A Case Report

**DOI:** 10.3390/jpm14030242

**Published:** 2024-02-24

**Authors:** Tsukasa Kuwana, Kosaku Kinoshita, Minori Mizuochi, Jun Sato, Nobutaka Chiba, Takeshi Saito, Toru Imai

**Affiliations:** 1 Division of Emergency and Critical Care Medicine, Department of Acute Medicine, Nihon University School of Medicine, 30-1, Oyaguchi Kami-cho, Itabashi-ku, Tokyo 173-8610, Japan; kuwana.tsukasa@nihon-u.ac.jp (T.K.); orita.minori@nihon-u.ac.jp (M.M.); sato.jun@nihon-u.ac.jp (J.S.); chiba.nobutaka@nihon-u.ac.jp (N.C.); saitou.takeshi@nihon-u.ac.jp (T.S.); 2 Department of Pharmacy, Nihon University Itabashi Hospital, Tokyo 173-8610, Japan; imai.toru@nihon-u.ac.jp

**Keywords:** blood concentration, case report, dextromethorphan, lipid sink, oral overdose

## Abstract

Dextromethorphan (DXM) is used to treat colds and coughs; however, it can cause central nervous system symptoms, such as severe serotonin syndrome (SS). To our knowledge, there is no specific treatment for severe DXM poisoning, and there are no reports on the clinical use of intravenous lipid emulsion (ILE) for its treatment. Herein, we report a case of severe DXM poisoning with SS that was successfully treated with ILE. An older adolescent male visited the emergency department 1 h after ingesting 4500 mg of DXM orally. Physical examination revealed generalized convulsions, muscle rigidity, mydriasis (8.0/8.0 mm), and flushed skin, with a Glasgow Coma Scale score of 8 (E3V1M4). Severe DXM poisoning with SS was diagnosed. The patient was intubated and administered midazolam for continuous convulsions and SS. Activated charcoal was also administered, and body surface cooling was performed. After an 11 h intensive care unit admission, SS with mydriasis (6.0/6.0 mm) did not improve. Subsequently, 1100 mL of 20% soybean oil was injected as an ILE. Mydriasis improved (3.5/3.5 mm) 30 min after ILE administration; simultaneously, blood DXM concentration rapidly increased approximately two-fold. After discontinuing midazolam, the patient’s consciousness signs improved, and he was weaned off the ventilator. SS was cured with no recurrence of convulsions. In cases of DXM poisoning with severe central nervous system disorders, such as SS, ILE treatment can potentially be an effective therapeutic option. For oral overdose cases, where the drug may remain in the intestinal tract, measures such as administering activated charcoal should be taken before administering ILE.

## 1. Introduction

Dextromethorphan (DXM) is an N-methyl-D-aspartic acid (NMDA) receptor antagonist used worldwide to treat colds and coughs as an over-the-counter drug. Owing to the effects of DXM on the central nervous system, such as euphoria, hyperexcitability, and hallucinations, DXM overdose has become a common problem, especially in adolescents [1]. Overdosing on DXM can lead to central nervous system disorders, such as severe serotonin syndrome (SS), which is characterized by mydriasis, semiconsciousness, tachycardia, tachypnea, marked sweating, ocular clonus, and unstimulated clonus in extremities [2]. SS has been documented to occur upon the administration of a combination of DXM and some antidepressants, such as selective serotonin reuptake inhibitors, serotonin–norepinephrine reuptake inhibitors, and other serotonergic medications [3]. However, reports of SS solely attributable to DXM overdose are scarce [2].

SS is caused by the overstimulation of serotonin receptors. The serotonin transporter maintains low serotonin concentrations and is important for the reuptake of the neurotransmitter into presynaptic nerve terminals. DXM has been suggested to inhibit the reuptake of serotonin by inhibiting the serotonin transporter. Consequently, this phenomenon increases the plasma and synaptic cleft concentrations of serotonin, which activate the serotonin receptors, ultimately causing SS [4]. Five fatal cases of DXM poisoning, two of which showed cerebral edema at autopsy, have been reported [5]. Although there have been some case reports of SS caused by DXM overdose or co-administration with antidepressants, there is currently no specific treatment for severe DXM poisoning, with symptomatic treatment being the primary approach [6,7,8]. Intravenous lipid emulsion (ILE) is reportedly effective against lipid-soluble drug poisoning [9], particularly in cases of local anesthetic systemic poisoning [10]. To our knowledge, there have been no reports on the clinical use of ILE for severe DXM poisoning; however, we report a survival case of DXM poisoning with severe SS, in which mydriasis improved immediately after ILE administration.

## 2. Detailed Case Description

An older adolescent male with a history of depression but not taking antidepressant medication regularly ingested 4500 mg of over-the-counter DXM pills (Medicon) and 6 mg of flunitrazepam orally in a suicide attempt. After overdosing, the patient experienced breathing difficulties and independently called for an ambulance. His vital signs on arrival of the ambulance were as follows: Glasgow Coma Scale (GCS) score, 14 (E4V4M6); heart rate (HR), 132 beats/min; respiratory rate (RR), 36 breaths/min; axillary temperature, 36.2 °C; arterial oxygen saturation (SaO_2)_ level, 100% (10 L/min, reservoir mask); and blood pressure, 142/80 mmHg. His pupils were mydriatic (5.0/5.0 mm), and the light reflexes in both eyes were brisk. Venous blood gas analysis on arrival revealed the following: pH, 7.247; PvCO_2_, 49.0 mmHg; HCO^3−^, 22.7 mmol/L; base excess, −9.5 mmol/L; and lactate, 4.5 mmol/L. He was transported to our hospital 1 h after the occurrence of the overdose incident. His vital signs on hospital arrival were as follows: GCS score, 8 (E3V1M4); HR, 139 beats/min; RR, 34 breaths/min; axillary temperature, 36.7 °C; SaO_2_ level, 97% (10 L/min, reservoir mask); and blood pressure, 70/58 mmHg. His pupils were mydriatic (8.0/8.0 mm), and the light reflexes in both eyes were brisk. An intravenous extracellular fluid bolus was quickly administered, owing to a decrease in blood pressure. The blood pressure increased within 30 min without continuous catecholamine administration and did not decrease afterward. Axillary temperature rose to 40.0 °C within 30 min, presenting with chills and shivers, and an increase in RR to 36 breaths/min. He exhibited generalized convulsions, muscle rigidity, and flushed skin. He met the Hunter Serotonin Toxicity criteria and was diagnosed with SS [11]. Arterial blood gas analysis after 1 h of arrival revealed the following: pH, 7.064; PaCO_2_, 80.8 mmHg; PaO_2_, 71.4 mmHg (room air); HCO_3_^−^, 22.7 mmol/L; base excess, −9.5 mmol/L; and lactate, 4.5 mmol/L. Owing to CO_2_ retention, disturbance of consciousness, and generalized convulsions, he was intubated and admitted to the intensive care unit (ICU). Activated charcoal was administered using a nasogastric tube after 1 h of arrival to counteract DXM poisoning. Although diazepam was administered for generalized convulsions, the convulsions recurred, prompting continuous administration of midazolam. The PaCO_2_ and pH quickly normalized after mechanical ventilation, arterial blood gas analysis indicated the following: pH, 7.360; PaCO_2_, 42.0 mmHg; PaO_2_, 118.0 mmHg (F_I_O_2_ 0.4); HCO_3_^−^, 24.2 mmol/L; base excess, −2.3 mmol/L. His hemodynamics also improved rapidly with the extracellular fluid infusion, resulting in a blood pressure of 122/52 mmHg and HR of 99 beats/min.

**Figure 1:**
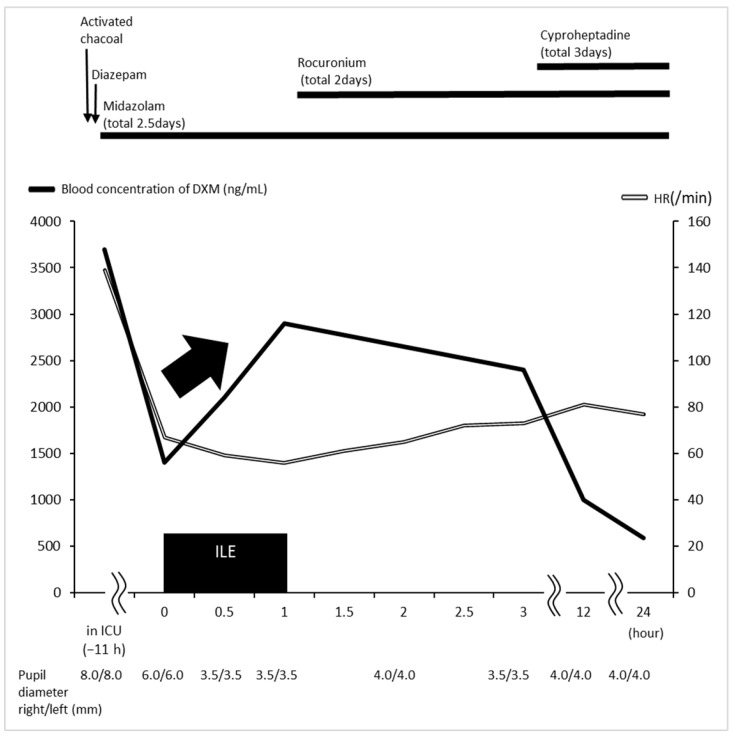
Timeline of case progression and blood DXM concentration, starting at the hour of ICU admission (11 h from ILE initiation). Mydriasis improved 30 min after ILE administration, and blood DXM concentration levels transiently increased and then decreased. DXM, dextromethorphan; HR, heart rate; ICU, intensive care unit; ILE, intravenous lipid emulsion.

After 11 h of ICU admission, there was no improvement in muscle rigidity, mydriasis (6.0/6.0 mm), hyperthermia (38.4 °C), or flushed skin owing to SS and disturbance of consciousness. In total, 1000 mL (100 mL bolus and 900 mL/1 h continuous administration) of 20% soybean oil injection (Intralipos injection 20%; Otsuka Pharmaceutical Factory Inc., Tokyo, Japan) was administered as ILE 12 h after oral intake of DXM. After 30 min of ILE administration, the pupils normalized (3.5/3.5 mm), and the light reflex of both eyes was brisk. After 12 h ICU admission, hypothermia therapy was initiated at 34.0 °C for 24 h using continuous rocuronium administration and rewarming to 36.0 °C for another 24 h. After 15 h ICU admission, cyproheptadine (32 mg/day) was administered via a nasogastric tube for SS. On the second day, the flushed skin improved. On the third day, after cessation of rocuronium and continuous administration of midazolam, consciousness improved to GCS score 15 (E4V5M6), and no recurrence of seizures was observed. The patient was then weaned off the ventilator. Subsequently, no recurrence of neurological symptoms such as mydriasis was observed. In addition, no recurrence of muscle rigidity, hyperthermia, and flushed skin due to SS was observed during hospitalization. On the seventh day, the patient’s vital signs were stable, with a GCS score of 15 (E4V5M6); HR of 82 beats/min; RR of 16 breaths/min; axillary temperature of 36.3 °C; SaO_2_ level of 96% (room air); and blood pressure of 124/60 mmHg. His pupils were mydriatic (2.0/2.0 mm), and the light reflexes in both eyes were brisk. Consequently, the patient was transferred from the ICU to another psychiatric hospital because of residual suicidal ideation. No ILE-induced liver injury, pancreatitis, lung injury, thrombosis, or blood clotting disorders were observed during hospitalization. Figure 1 shows the timeline of case progression and blood DXM concentration starting at the hour of ICU admission (hours). Blood DXM concentrations were as follows: −11 h (on ICU admission), 3700 ng/mL; 0 h (immediately before ILE), 2100 ng/mL; 1 h, 2900 ng/mL; 3 h, 2400 ng/mL; 12 h, 1000 ng/mL; and 24 h, 590 ng/mL. Blood DXM concentration approximately doubled after 1 h of ILE administration, with simultaneous improvement in mydriasis. Thereafter, blood DXM concentration gradually declined. Written informed consent to publish case details was obtained from the patient.

## 3. Discussion

ILE is a potentially beneficial therapeutic option for severe central nervous system disorders, including SS, caused by drug poisoning, such as DXM. DXM is highly penetrable into the brain. In rats, plasma DXM correlated with brain DXM, and the brain/plasma concentration ratio was approximately 6.5 [12]. Therefore, in the case of DXM overdose, it is speculated that central nervous system toxicity, such as SS, will persist for a long period. In this case study, central nervous system symptoms, such as mydriasis and disturbed consciousness, persisted for 12 h after oral administration (11 h after ICU admission), suggesting that DXM distribution in the central nervous system causes central nervous system toxicity. However, after ILE administration, blood DXM concentration transiently doubled within an hour, with a simultaneous improvement in mydriasis, and then decreased. This transient increase in blood DXM was consistent with the lipid sink mechanism of redistribution of the causative drug from tissues to the blood [13]. In an animal study of the relationship between blood amitriptyline levels and tissue concentrations after ILE administration, the blood amitriptyline levels increased in the ILE groups compared with those in the non-ILE groups, and its concentration decreased in the brain and heart [14]. In addition, intravenous injection of bupivacaine in rats followed by ILE administration decreased the bupivacaine content in the brain, heart, lungs, kidneys, and spleen, as a lipid sink phenomenon [15]. These reports indicate that ILE retransfers the drug concentrations in tissues, such as the brain, into the blood. These phenomena are consistent with the lipid sink mechanism. In addition, these studies demonstrate that the effects of ILE are immediate. Another clinical case report of intractable seizures due to amoxapine poisoning showed that the seizures ceased immediately after ILE administration [16]. In this case study, blood DXM concentration increased rapidly immediately after ILE administration, and mydriasis, a symptom of SS, improved simultaneously. This suggests that the lipid sink mechanism transferred DXM into the blood from the central nervous system. The use of ILE should be considered with the persistence of central nervous system symptoms, such as SS; however, the use of ILE should not be determined solely based on the blood concentration level.

The octanol/water (O/W) partition coefficient, which indicates drug lipid solubility, is generally considered effective for ILE at values ≥logP of 2. Notably, the magnitude of the reduction in blood concentrations of intoxicating drugs is positively correlated with fat solubility [17]. Lidocaine causes local anesthetic poisoning, and ILE is frequently used to treat lidocaine toxicity [18]. Lidocaine is highly lipid-soluble, with an O/W partition coefficient of logP = 2.39 [19]. However, DXM is not highly lipid-soluble, with an O/W partition coefficient of logP = 1.23 [20]. Although DXM is not so highly lipid-soluble, blood DXM concentration rapidly doubled after ILE administration with a simultaneous improvement in SS-induced mydriasis, suggesting that ILE could be a useful therapeutic option for severe central nervous system disorders, such as SS, even if its lipid solubility is not so high. In addition, ILE has been reported to exhibit an effect on various drug addictions within a remarkably short time frame, often within minutes of administration. In QRS duration prolongation due to amitriptyline cardiotoxicity in amitriptyline overdose, QRS width improved within minutes after ILE administration [21]. In another case report, ILE was administered for premature ventricular contractions due to local anesthetic intoxication, and the PVCs resolved quickly [22]. The onset time of the effect of ILE in this case report is consistent with these previous reports. However, as a limitation of this study, DXM brain concentrations were not measured in this case; therefore, it is unclear whether there was a direct relationship between DXM brain concentrations and SS symptoms. Further investigation is warranted to elucidate the detailed mechanism of the therapeutic effect of ILE in SS.

To exercise caution when administering ILE, if the drug remains in the intestinal tract in case of an oral overdose, drug absorption may be accelerated using ILE, as demonstrated in rodent models, suggesting that ILE injection can enhance the absorption of lipid-soluble drugs from the gastrointestinal lumen. Early administration of ILE after 30 min of oral amitriptyline and verapamil ingestion was associated with increased mortality [23]. In addition, early death after ILE administration owing to rectal thiopentone overdose has been reported in rat models [24]. Therefore, Cave et al. [25] suggested that ILE should not be administered in the early stages of life-threatening oral overdose, and measures such as activated charcoal administration should be taken before administering ILE. In our case, the favorable outcome could be attributed to the administration of activated charcoal immediately after the overdose and the administration of ILE 12 h after oral overdosing, when the drug had already been absorbed from the intestinal tract. 

This case report has some limitations. First, an improvement in acid–base equilibrium was observed following ventilator management prior to ILE administration. Furthermore, hemodynamic stabilization was observed over time with extracellular fluid infusion before ILE administration. Therefore, the improvement in acid–base equilibrium and hemodynamic stabilization prior to ILE administration may have contributed to the improvement in central nervous system symptoms. Furthermore, the continuous administration of midazolam to prevent the recurrence of convulsions before ILE administration, and the initiation of hypothermia therapy after ILE administration may have also contributed to the improvement in central nervous system symptoms. In addition, cyproheptadine was administered to address SS following ILE administration. Thus, it may be difficult to establish the effect of ILE alone, given that the patient received other treatments at different times. Nevertheless, the temporal correlation observed between ILE administration and the improvement in mydriasis in the present case suggests the potential therapeutic efficacy of its use in DXM poisoning cases.

Overall, this case exemplifies the usefulness of lLE in the management of DXM poisoning. ILE appeared to induce rapid alleviation of CNS symptoms, particularly SS-associated manifestations, including pupillary diameter in DXM poisoning. This effect probably prevented further destabilization of the patient’s clinical course. In situations where there may be drug residues in the intestinal tract, preemptive measures such as administration of activated charcoal via a nasogastric tube should be taken before administering ILE. With the increasing prevalence of DXM poisoning worldwide, which leads to fatal or severe outcomes, as observed in the present case, ILE has emerged as a valuable adjunctive treatment option for severe DXM poisoning, owing to its favorable safety profile in terms of side effects, low cost, and ease of administration.

## 4. Conclusions

ILE was administered 12 h after oral intake of DXM in a survival case of severe DXM overdose with SS. Therefore, ILE could be a useful therapeutic option for severe central nervous system symptoms, such as SS, caused by drug poisoning.

## Data Availability

Patient data for this case report are stored in the electronic medical records of Nihon University Hospital.
